# Nasopharyngeal Polyp in a Patient With Submucous Cleft Palate

**DOI:** 10.7759/cureus.14787

**Published:** 2021-05-01

**Authors:** Mosaad Abdel-Aziz, Gamal Abdel-Fattah, Nada M Abdel-Aziz

**Affiliations:** 1 Otolaryngology - Head and Neck Surgery, Cairo University, Cairo, EGY; 2 Otolaryngology - Head and Neck Surgery, Misr University for Science and Technology (MUST), Cairo, EGY; 3 Pediatric Dentistry, October University for Modern Sciences and Arts (MSA), Cairo, EGY

**Keywords:** nasal septal polyp, nasopharyngeal polyp, submucous cleft palate, hypernasality, pharyngeal flap

## Abstract

Encountering a nasopharyngeal polyp in a patient with submucous cleft palate (SMCP) is a difficult problem, as the lesion could support the weak palate. Removal of this lesion may unmask the SMCP with consequent worsening of speech nasality. Nasal septal polyp protruding to the nasopharynx in a patient with SMCP has not been reported before in the literature. This report describes a septal polyp arising from the posterior border of the nasal septum and protruding in the nasopharynx in a 16-year-old girl with submucous cleft palate. The polyp appeared to support the weak palate, and they acted as a ball and socket during speech articulation. Removal of this polyp may result in velopharyngeal insufficiency. Trans-nasal endoscopic removal of the polyp with obturation of the velopharyngeal port with a superiorly-based pharyngeal flap was performed in the same sitting. Pre- and postoperative speech evaluation using auditory perceptual assessment and nasometry revealed no worsening of nasality, also the patient reported improvement of her nasal breathing. We concluded that, the presence of a nasopharyngeal polyp in a patient with SMCP may compensate the speech problem. Removal of the polyp and treatment of SMCP by a pharyngeal flap in one-sitting is an effective procedure without adverse effect on patient’s speech.

## Introduction

A submucous cleft palate (SMCP) is characterized by lateral diversion of palatal musculature insertion leaving the central midline part of the soft palate deficient of muscles [1]. It is usually diagnosed by three criteria which are bifid uvula, bluish mucosa of the midline of the soft palate, and notched posterior part of the hard palate that can be felt by palpation [2,3]. A patient with SMCP may present with velopharyngeal dysfunction. Hypertrophied adenoid as a space occupying nasopharyngeal tissue may support the weak palatal musculature, however, this supportive effect may be diminished with adenoid involution resulting in speech hypernasality [4].

The nasal septum is a very rare site of origin for nasal polyps that usually arise from the sinus mucosa and protrude to the nasal cavity through the ostia of the sinuses [5]. However, nasal septal polyp has been previously reported as a rare case report in the literature [5-7]. Here, we present a 16-year-old girl with nasal septal polyp that caused nasal obstruction with SMCP.

## Case presentation

**Figure 1:**
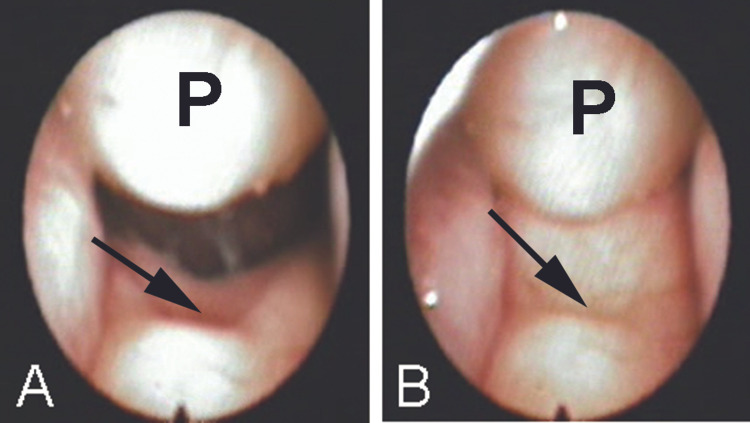
Preoperative flexible nasopharyngoscopic views Preoperative flexible nasopharyngoscopic views showing the nasopharyngeal polyp (P) and the arrow pointing to a notched soft palate during breathing (A), and during speech articulation (B). The polyp and the palate appear as a ball and socket.

A 16-year-old girl with a history of bilateral nasal obstruction for four years was admitted. The complaint progressively worsened over time. The patient had no history of allergic rhinitis or rhinosinusitis, however, her parents referred of hypernasality of their girl’s speech dating since articulation development with improvement in the last three years. Endoscopic rigid nasal examination revealed a large nasopharyngeal polypoid mass originating from the posterior border of nasal septum. Otoscopic examination revealed no abnormality of both ears. Oral examination showed criteria of SMCP [2,3], so, flexible nasopharyngoscopy was performed as a routine examination for patients with palatal abnormality. The midline of the nasopharyngeal surface of the soft palate was seen concave indicating deficient palatal muscles, and a large nasopharyngeal polyp was seen occupying this concavity that looked like a ball and socket on speech articulation (Figure 1).

**Figure 2:**
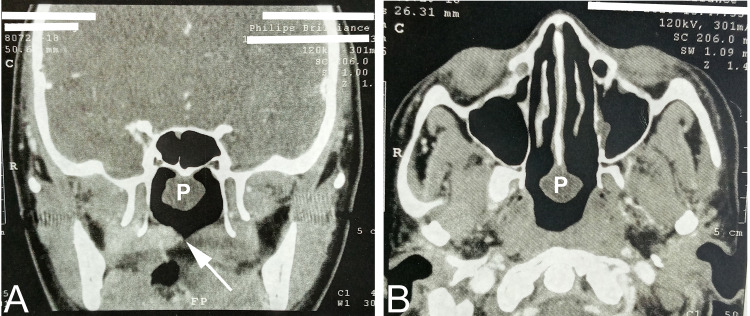
Preoperative computed tomographic views for the nasopharynx (A) A coronal view shows a polyp (P) in the upper part and the arrow points to a notched soft palate in the lower part of the nasopharyngeal cavity. (B) An axial view shows the polyp (p) originating from the posterior border of the nasal septum.

Computed tomography (CT) showed a large nasopharyngeal mass originating from the posterior border of the nasal septum with deficient midline palatal muscles (Figure 2).

We planned to remove the polyp endoscopically, a procedure that may adversely affect the speech, so, we also planned to obturate the velopharyngeal port with a superiorly-based pharyngeal flap. Speech evaluation was performed using auditory perceptual assessment (APA) and nasometric assessment. APA included assessment of hypernasality, nasal air emission and weak pressure consonants, then we graded each item on a 5-point scale (0-4) in which 0 means normal and 4 means severe affection. Nasalance score (NS) was measured using a Nasometer (Model 6200; Kay Elemetrics Corp., Lincoln Park, NJ). The nasometric data were obtained while the patient repeated standardized nasal and oral sentences.

Under general anesthesia, nasal endoscopic removal of the polyp was performed followed by cauterization of its pedicle on the posterior border of the nasal septum. On the same sitting, a superiorly-based midline pharyngeal flap was elevated from the posterior pharyngeal wall, and it was inserted in the soft palate midway between the posterior border of the hard palate and the posterior border of the soft palate through a transverse palatal split [1]. This technique was performed to obliterate a potential defect that may occur postoperatively. No intraoperative or postoperative complications were encountered. Histopathologic examination of the lesion revealed that it was an inflammatory polyp. The patient reported improvement of her nasal breathing without noticeable speech affection.

**Figure 3:**
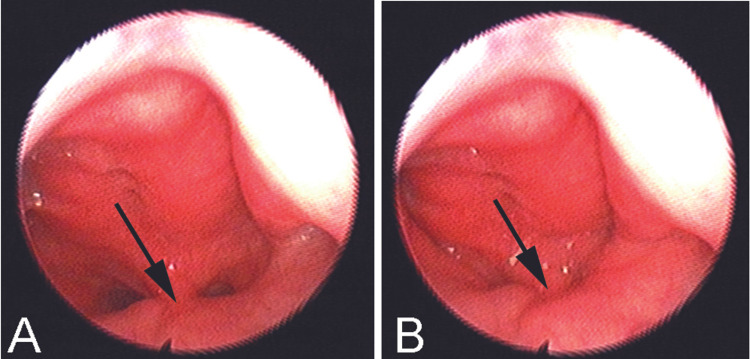
Postoperative flexible nasopharyngoscopic views Postoperative flexible nasopharyngoscopic views show the velopharyngeal port and the arrow points to the pharyngeal flap in the mid-line during breathing (A) and during speech articulation (B).

Three months postoperatively, speech was evaluated using the same parameters employed preoperatively, then we compared both preoperative and postoperative results. The preoperative APA score was 5 that improved to 4 postoperatively. The preoperative NS was 35 for nasal sentences and 13 for oral sentences, improved to 32 for nasal sentences and 11 for oral sentences. Also, flexible nasopharyngoscopy showed complete closure of the velopharyngeal port during articulation of speech (Figure 3).

Nonetheless, the patient was advised to receive speech therapy as a routine method after surgical treatment of velopharyngeal insufficiency.

## Discussion

Encountering a nasopharyngeal polyp in a patient with SMCP is a difficult problem, as the lesion could support the weak palate. Removal of this space occupying nasopharyngeal lesion may unmask the SMCP with consequent affection of speech. Children with SMCP may complain of speech hypernasality especially after adenoid involution [4]. The same problem was expected to occur in our presented case after removal of the nasopharyngeal polyp. The parents of our patient reported that girl’s speech hypernasality had been improved spontaneously three years before presentation to us. Improvement of speech nasality may be explained by the development of this nasopharyngeal polyp which could have a compensatory effect supporting the weak palate.

We treated our patient with a superiorly-based pharyngeal flap in order to compensate the velopharyngeal gapping that may occur after removal of the nasopharyngeal polyp. As the speech may be affected by the procedure, we assessed the speech before and three months after intervention. We detected no worsening effect of speech nasality postoperatively relative to the preoperative values. Also, the velopharyngeal port was seen competent on speech articulation. On the other hand, the patient reported subjective improvement of nasal breathing.

SMCP could be treated with reconstruction of the palatal muscles either using Furlow double-opposing z-plasty, or intravelar veloplasty [4]. However, a pharyngeal flap can be used especially in patients with thin palatal muscles to avoid the need of a secondary corrective surgery if palatal reconstruction fails [1]. Indeed, pharyngeal flap surgery is usually superior to palatal reconstruction procedures on the velopharyngeal closure, since it has more obstructive impact on the airway [8]. In our case, the patient reported improvement of her nasal breathing postoperatively.

The source of nasal polyps is usually the paranasal sinuses, so, they commonly protrude through the lateral nasal wall to the nasal cavity [5]. Nasal septum has been rarely reported as a source of polyps in the literature [5-7]. Stefano et al. [6] described the lesion in two patients who had nasal septal deviation, while Akdogan et al. [7] detected the lesion bilaterally in one patient originating from the superior part of the nasal septum and affecting the olfactory function. Also, it has been reported to extend posteriorly to the nasopharynx through the choana, and it was termed as a septochoanal polyp [9,10]. Nevertheless, in our studied patient the polyp originated from the posterior border of the nasal septum and prolapsed in the nasopharynx. Histopathologic examination revealed its inflammatory nature. It may be caused by mucosal inflammatory reaction secondary to fluid regurgitation as a result of SMCP. Ozgirgin et al. [5] and Stefano et al. [6] suggested the cause of this rare lesion to be inflammatory. Fortunately, the occurrence of this polyp in our patient with SMCP may cause palatal support and consequently decrease in speech hyper-resonance. So, we considered the decision of velopharyngeal obturation concomitantly with polyp removal to avoid velopharyngeal dysfunction postoperatively.

## Conclusions

The presence of a nasopharyngeal polyp in a patient with SMCP could compensate the velopharyngeal gap. Polyp removal and treatment of SMCP by a pharyngeal flap in one-sitting is an effective procedure without adverse effect on patient’s speech.
